# Generating Evidence from Expanded Access Use of Rare Disease Medicines: Challenges and Recommendations

**DOI:** 10.3389/fphar.2022.913567

**Published:** 2022-05-23

**Authors:** Tobias B. Polak, David G. J. Cucchi, Joost van Rosmalen, Carin A. Uyl-de Groot, Jonathan J. Darrow

**Affiliations:** ^1^ Erasmus School of Health Policy and Management, Erasmus University Rotterdam, Rotterdam, Netherlands; ^2^ Department of Biostatistics, Erasmus University Rotterdam, Rotterdam, Netherlands; ^3^ Department of Epidemiology, Erasmus University Rotterdam, Rotterdam, Netherlands; ^4^ Real-World Data Department, myTomorrows, Amsterdam, Netherlands; ^5^ Department of Internal Medicine, Franciscus Gasthuis & Vlietland, Rotterdam, Netherlands; ^6^ Department of Hematology, Cancer Center Amsterdam, Vrije Universiteit Amsterdam, Amsterdam University Medical Center, Amsterdam, Netherlands; ^7^ Department of Law and Taxation, Bentley University, Waltham, MA, United States; ^8^ Division of Pharmacoepidemiology and Pharmacoeconomics, Department of Medicine, Brigham and Women’s Hospital and Harvard Medical School, Boston, MA, United States

**Keywords:** expanded access, compassionate use, rare disease, drug regulation, real-world data, perspective, policy recommendation

## Abstract

Patients with rare diseases often have limited or no options for approved treatments or participation in clinical trials. In such cases, expanded access (or “compassionate use”) provides a potential means of accessing unapproved investigational medicines. It is also possible to capture and analyze clinical data from such use, but doing so is controversial. In this perspective, we offer examples of evidence derived from expanded access programs for rare diseases to illustrate its potential value to the decision-making of regulators and payers in the European Union and the United States. We discuss ethical and regulatory aspects to the use of expanded access data, with a focus on rare disease medicines. The heterogeneous approach to expanded access among countries within the European Union leaves uncertainties to what extent data can be collected and analyzed. We recommend the issuance of new guidance on data collection during expanded access, harmonization of European pathways, and an update of existing European compassionate use guidance. We hereby aim to clarify the supportive role of expanded access in evidence generation. Harmonization across Europe of expanded access regulations could reduce manufacturer burdens, improve patient access, and yield better data. These changes would better balance the need to generate quality evidence with the desire for pre-approval access to investigational medicine.

## Introduction

An estimated 7,000 rare diseases affect approximately 10% of the population^1^. Although the number of patients with a given rare disease is by definition limited, the collective impact of these diseases is substantial. Yet only about one in 42 patients with a rare disease had even a single United States (US) Food and Drug Administration (FDA)-approved treatment option (Ferreira, 2019). Before granting marketing authorization, regulatory agencies require evidence that the treatment benefits outweigh the risks, and generating such evidence requires time. Patients who have neither time nor approved treatments at their disposal and are unable to participate in trials, may seek access to investigational medicines *via* expanded access programs (Darrow et al., 2015).

Expanded access pathways allow patients with life-threatening or debilitating conditions to access unapproved medicines. Terminology for expanded access programs varies, as in English alone it is known as “named-patient use,” “single-patient IND,” “compassionate use,” or as “expanded,” “managed,” “early” or “special” access, all to denote non-trial access to unlicensed medicine (Kimberly et al., 2017).

Historically, expanded access pathways were designed primarily to provide a treatment—to grant patients access to medicine outside of studies as last resort—although the collection of additional data was also contemplated (Office of the Federal Register National Archives and Records Administration, 1987). Over the years, there has been a shift to increasingly emphasize the role of expanded access data. Although the primary intent of expanded access remains providing treatment to patients, data generated through expanded access have been reported in a large number of peer-reviewed publications, submitted in regulatory filings to the FDA and the European Medicines Agency (EMA), and used in health technology assessments (Polak et al., 2022b). However, opinions differ regarding to what extent data can be collected in the first place, and if so, how and when such data can be relied upon.

In this perspective, we clarify issues of data collection and subsequent analysis during expanded access programs in the US and European Union (EU). We first discuss detailed examples from the usage of expanded access data relating to rare disease medicines. Subsequently, we highlight the discrepancies in regulatory views on expanded access, discuss related issues of access inequality, and finally discuss ethical considerations of data collection and analysis. Lastly, we suggest means for improving expanded access data collection and use, with a particular focus on the EMA.

## Reporting and Use of Expanded Access Data

### Reporting of Expanded Access Data in Peer-Reviewed Publications

Expanded access has recently gained attention by the large number of compassionate use studies or case reports on treatments for SARS-CoV-2, such as remdesivir and convalescent plasma (Grein et al., 2020; Joyner et al., 2021). Unpublished data from our group indicate that from 2000 to 2022 over 1,300 expanded access studies have been published. In oncology an estimated 198 expanded access studies were published with several examples concerning rare diseases from 2013 through 2020 (Borysowski et al., 2021). The median number of patients in publications that were not case reports (80%) was 153. This number ranged from N = 7 in a publication reporting the experience of Austrian physicians using venetoclax to treat high-risk patients with acute myeloid leukemia refractory to standard therapy, to N = 4,543 patients from over 50 countries in a report of the expanded access program for sunitinib to treat metastatic kidney cancer (Gore et al., 2015; Huemer et al., 2019). Both sunitinib^2^ and venetoclax^3^ received orphan designation for these diseases by the EMA.

Several drugs are associated with numerous publications flowing from expanded access, such as cabazitaxel, a chemotherapeutic for metastatic, castration-resistant prostate cancer (de Bono et al., 2010). It is associated with at least 10 expanded access studies, separately reporting experiences in Spain, Australia, Germany, South-Korea, Naples, Italy, the Netherlands, Canada, United Kingdom, and Europe (Wissing et al., 2013a, 2013b; Di Lorenzo et al., 2013; Heidenreich et al., 2013, 2014; Bracarda et al., 2014; Castellano et al., 2014; Lee et al., 2014; Parente et al., 2017; Yokom et al., 2018). The outcomes measured in these reports are heterogeneous, ranging from only safety data, to data on safety and quality-of-life, to data on safety and effectiveness, while others focus on prognostic modelling (Wissing et al., 2013b; Bracarda et al., 2014; Lee et al., 2014; Yokom et al., 2018). The heterogeneous reporting of different outcomes, and the multiplicity of reports across countries indicate the lack of harmonization or best practices in this setting.

### Use of Expanded Access Data in Regulatory Filings

Regulators require the conduct of clinical trials to determine safety and efficacy before granting marketing authorization. For rare diseases, performing such trials can be slow due to low patient enrolment, or even unfeasible or unethical (Berger et al., 2017). Therefore, any evidence generated through expanded access patients should be harnessed to help clarify harms and benefits.

Through 2018 and starting in 1955 (FDA) or 1995 (EMA), 49 drug-indication pairs were approved by either the EMA or FDA based in part or in whole on expanded access data, 31 (63%) of which had an “orphan designation” to support the development and evaluation of treatments for rare diseases (Polak et al., 2020a). This includes for example lutetium-177 oxodotreotide, a radioactive treatment for gastroenteropancreatic neuroendocrine tumors. Supplementary to the pivotal randomized controlled trial (N = 229), data from 558 patients treated under compassionate use were considered in support of the indication. In the case of cholic acid, a treatment for patients suffering from various rare genetic disorders in bile acid metabolism, all evidence came from expanded access. The FDA and EMA evaluated data from two expanded access programs (N = 63, N = 22) to support the marketing authorization. The EMA approved cholic acid under exceptional circumstances, because

*“the applicant was unable to provide comprehensive data on the efficacy and safety of the medicine under normal conditions of use. This can happen because the condition to be treated is rare or because collection of full information is not possible or is unethical”* ^4^ *.*

In the 39 cases where expanded access programs were included in the “pivotal efficacy section” of regulatory submissions for rare disease medicines, 58% of all patients were treated under expanded access pathways (Polak et al., 2020a). Expanded access data can also be used to obtain special regulatory designations: in 2014, the FDA granted “breakthrough designation” to uridine triacetate based on published case studies and expanded access data (Ison et al., 2016). This highlights the role of expanded access in regulatory decision making in rare diseases.

### Use of Expanded Access Data in Health Technology Assessments

As expanded access programs may provide the first source of evidence on the treatment use of investigational medicine in non-trial populations, various countries have explicitly combined expanded access with evidence generation or reimbursement schemes, such as L’Accèss Précoce in France, the DRUG Access Protocol in the Netherlands and the Early Access to Medicines Scheme (EAMS) in the UK (Balasubramanian et al., 2016; Haute; Autorité de Santé, 2021; Polak et al., 2022a; Zeverijn et al., 2022).

In the United Kingdom (UK), drug approval is followed by a separate appraisal of cost-effectiveness compared to existing treatment options. 21% of the health technology assessments conducted for the National Health Service in the last decade have relied in part on expanded access data (Polak et al., 2022b). We here highlight ipilimumab, a treatment for advanced, previously treated, unresectable skin cancer, which was approved in 2011 based on a trial involving 676 patients. For ipilimumab, the number of vials of drug needed is based on patient weight. As only 55 patients from the UK participated in the pivotal trial, the addition of expanded access patients helped the reimbursement agency obtain a better estimate of vial usage in the real-world patient population in their jurisdiction. At the reimbursement stage, data were pooled from 258 UK patients receiving ipilimumab through an expanded access program (using 1.19 vials of 50 mg on average) to supplement the data from the pivotal regulatory trial (using 1.51 vials of 50 mg on average). In this particular case, including data from expanded access led to a decrease in mean cost estimates.

## Regulatory and Ethical Aspects

### The United States: Treatment or Research?

Despite the frequent use of evidence from expanded access programs, opinions differ on the extent to which data can be collected in this setting and in what way such data should be relied on. Expanded access pathways were first formalized by the US FDA in 1987 (Darrow et al., 2015). The focus was primarily on providing treatment: in a meeting on 14 January 1993, the National Institutes of Health discussed the “research” status of patients in US compassionate use programs for gene therapies (Chapman et al., 2019). An FDA staff member noted that:

*“The Office for Protection from Research Risk maintains that such patients cannot be considered research subjects. An investigator who receives a single patient compassionate use exemption cannot include the results of that patient data in any further reports of their research.”*

However, the current US legislation does not imply such a strict dichotomy between “research” and “treatment”—there even is no clarity to whether participants in expanded access programs should be considered patients or research subjects. In the US, the expanded access program occurs under an “investigational new drug application” and the dispensing physician is considered an “investigator^5^.” The main intent of expanded access programs—to provide treatment—is thus in tension with this regulatory framework, which generally views the purpose of an investigational new drug application to be the conduct of clinical trials, for which the primary intent is evidence generation. Over the years, expanded access has been increasingly viewed as an alternate means of collecting information on harms and benefits. In a 2020 conference, the FDA’s principal deputy commissioner Janet Woodcock explicitly confirmed the agency’s:

“*greater acceptance of data from (expanded access) treatment use to enhance generalizability in clinical development*” (Woodcock, 2020).

Although the views stated above are 27 years apart, there still is no consensus among regulators, bio-ethicists and drug developers on the ability to collect and analyze data from compassionate use (Bunnik et al., 2018; Polak et al., 2020b; Rozenberg and Greenbaum, 2020; Bunnik and Aarts, 2021; Kearns et al., 2021).

### The European Union’s Perspective

In the EU, individual member states regulate expanded access programs. Although the EMA governs marketing authorizations *via* a centralized procedure, the EMA has no formal authority over expanded access requests and plays only an advisory role. The regulatory reluctance to rely on data from expanded access programs stems from concern over data quality. In the Guideline on Compassionate Use of Medicinal Products from 2007, the EMA has dedicated a section titled “*compassionate use vs. clinical trials”* to address this issue:

“*From a methodological point of view, clinical trials are practically the only means of obtaining reliable and interpretable efficacy and safety data for a medicinal product. Although safety data may be collected during compassionate use programmes, such programmes cannot replace clinical trials for investigational purposes. Compassionate use is not a substitute for properly conducted trials”* (CHMP/EMEA/27170/2006, 2007).

But this section does not foreclose the use of expanded access data as a supplement to clinical trial data, rather than as a replacement for them. We are not aware of any evidence of companies or physicians bypassing trial guidelines and conducting expanded programs instead—some companies have refused expanded access requests to avoid jeopardizing trial enrollment^6^. Some worry, however, that allowing limited use of expanded access data could lead to increasing calls to broaden use of expanded access data. Illustratively, Belgian authorities describe a “Frequently Asked Question,” “*Could we apply for a Compassionate Use Program (CUP) or Medical Need Program (MNP) in place of an extension trial/open label study?”.* Such concerns have led some countries to prohibit data collection through sponsors on expanded access studies. In earlier versions of this FAQ, the Belgian authorities responded that:

*“no other data except pharmacovigilance data can be gathered which will only be used for the evaluation of the (..) program”* (Federal Agency for Medicines and Health, 2019).

This even precluded the use of safety data for purposes other than the evaluation of the expanded access program. In more recent versions, this has changed to:

“*data collected (…) that are necessary for the conduct of the program (e.g., to check inclusion/exclusion criteria, to follow-up the B/R (benefit/risk) of a patient, pharmacovigilance data) could be used to enlarge the understanding of the treatment. It is not possible to collect more data than strictly needed for the conduct and evaluation of the program”* (Federal Agency for Medicines and Health, 2022).

Similarly, Austria prohibits data collection in a named-patient setting (“*Heilversuch”*) stating that:

*“named patient use is intended to facilitate the urgently needed treatment of a specific patient to avert a life-threatening or chronically debilitating situation. Systematic collection of data on safety and efficacy of the medicinal product used is not legally acceptable in this framework”* (BASG, 2015).

Through our correspondence with regulators, we learned that Sweden does not allow data collection at all, and that Canada does not “condone” data collection. Nevertheless, several publications on expanded access programs originate from Austria, Sweden, Belgium, and Canada (Steger et al., 2005; Lyckegaard et al., 2007; Chen et al., 2009; Freedman et al., 2009; Bracarda et al., 2015; Winqvist et al., 2019; Servais et al., 2020; Schubert et al., 2021). These paradoxes demonstrate the unclear position of expanded access in evidence generation.

### Access Inequality

The current set-up of expanded access, in which individual EU member states retain full freedom to regulate these programs within their borders, forces companies to navigate a complex array of pathways that are often only accessible in local languages. Pharmaceutical companies without local presence or sufficient resources may prefer to provide access in countries with easier access pathways, raising issues of patient access equity.

The cost of expanded access creates further complications. Although manufacturers mainly provide treatment free-of-charge, France is willing to pay for treatment under expanded access, Italy has reimbursement options for expanded access in rare diseases, and the US allows the sponsor to recover the direct costs (e.g., manufacturing, shipment) from private or government payers. Most other countries prohibit paying for unlicensed medicine, or even charge the manufacturer for setting up an expanded access program. Belgium charges €19,835 to set-up a compassionate use program, and participation in the UK EAMS scheme comes at a fee of £25,643 (Federal Agency for Medicines and Health, 2022)^7^. These costs may discourage pharmaceutical companies from participating in expanded access programs, negatively impacting patient access.

### Ethical Implications

Providing treatment without collecting relevant data deprives future patients of the benefit of known outcomes and denies the patient the opportunity to altruistically contribute to generalizable knowledge. Prohibiting the use and collection of data could reduce manufacturer willingness to provide expanded access, affecting even those countries that allow or encourage such reliance.

Furthermore, expanded access is non-randomized and unblinded, which can lead to confounding (Polak et al., 2020b; Rozenberg and Greenbaum, 2020). There are no guidelines on the quality assurance of data collection in expanded access—Good Clinical Practice is mandated by the EMA only for interventional trials (European Parliament, 2014). Regulators or ethics committees should therefore ensure that expanded access does not undermine enrolment in traditional clinical trials adequate to generate high-quality evidence. The recent US convalescent plasma expanded access program for SARS-CoV-2 showed that this fear is not unfounded. Over 105,717 patients were enrolled in this program before trials where fully enrolled or completed (Yang et al., 2021). Although a first analysis of these single-arm data hinted at beneficial treatment effects, randomized trials later did not confirm that convalescent plasma improved outcomes in inpatient care (Janiaud et al., 2021; Joyner et al., 2021).

Lastly, it should be carefully determined whether the benefits of evidence generation outweigh the additional paperwork and research strains imposed on patients and physicians—the changing nature of compassionate use programs to contribute “research” in addition to “treatment” has posed concerns to bioethicists (Chapman et al., 2019). Ethical oversight could ensure that data collection respects the treatment intent of expanded access.

## Policy Recommendations

In this perspective we have illustrated the usage of expanded access data in rare disease medicines in scientific publications, regulatory filings, and health technology assessment. Although these data are frequently used, the role of expanded access in evidence generation, and the regulations governing data collection, are extremely divergent. The European set-up of compassionate use is a patchwork of national access pathways, which may deter rather than expedite patient access to investigational medicine. We here offer several potential policy recommendations.

First, we call for regulatory guidance for data collection in expanded access settings, for example by including expanded access in real-world evidence frameworks, or offering means of integrating expanded access data in the guideline on patient registries (CHMP/EMEA, 2021). This guidance should acknowledge the observational nature, suggest means for assuring data quality (remote monitoring, database requirements), and ensure that the burden placed on physicians and patients for data collection is justified by the needs for additional evidence generation. Lastly, it could highlight the types of data collection that may be most desirable, such as real-world patient demographics, dosing, or treatment adherence. For rare diseases, a more flexible approach regarding the use of expanded access data could be considered.

Second, the EMA guidelines could be revised to encourage the responsible use of expanded access data. Guidelines could clarify that expanded access data cannot replace clinical trial data, but may supplement such data to inform usage in non-trial populations or to increase patient numbers in rare disease. This is consistent with other efforts to expand use of “real-world evidence,” or evidence derived from non-trial data sources (Sherman et al., 2016). The lack of mention of efficacy data by the EMA is not in line with individual member states’ initiatives that explicitly combine expanded access and evidence generation. Such paradoxes should be prevented and a future revision of the guidelines should include efficacy outcomes.

The notion that clinical trials are the only means of obtaining reliable information does not align with the inclusion of expanded access data in decision making by the EMA: regulatory submissions have included data from expanded access programs to clarify the efficacy and safety profile of certain drugs (Polak et al., 2020a). The EMA guideline from 2007 discusses expanded access “versus” clinical trials, which is at odds with the recently stated vision of EMA executive director Emer Cooke who argued:

*We believe that the binary discussion between clinical trials and RWE is unhelpful as each approach brings its own strengths and weaknesses* (Arlett et al., 2022).

The historical distinction between “research” and “treatment” intent is not always clear—nor should this imply that the primary intent (treatment) should prevent other (research) usages. Electronic health records are clearly intended to aid in the treatment of patients, but have been harnessed on a grand scale to simultaneously facilitate research (Desai et al., 2021).

Third, the conduct of multinational observational studies warrants simplification. The European Clinical Trial Regulation expedites interventional studies *via* a shared assessment by member states. For non-interventional studies no such pathway exists, which hinders the set-up of studies. This potentially explains why publications frequently cover only the national experience within international compassionate use programs. The burden of setting up separate studies within each individual country or region affects rare diseases in particular, where the effort of initiating an observational study may not outweigh the limited data collection benefits. A centralized non-interventional study procedure could resolve these issues.

Fourth, we call for the creation of a unified EU expanded access pathway. The main goal of compassionate use is to provide “early access” to investigational medicine for patients in need. The current set-up consists of a non-binding, optional advice procedure from the EMA, as well as 27 member states with multiple different pathways per member state. To provide expanded access, some countries require ethics committee approval, others do not. Some countries pay for treatment cost, others demand fees from manufacturers. Some countries allow liberal data collection, while others do not allow data collection at all. Harmonization and standardization of compassionate use pathways could reduce costs to regulators and manufacturers and resolve issues of equity in patient access, while also facilitating data collection to supplement trial data, which can be especially important for patients with rare diseases.

## Data Availability

The original contributions presented in the study are included in the article, further inquiries can be directed to the corresponding author.

## Footnotes

1
https://rarediseases.org/.

2
https://www.ema.europa.eu/en/medicines/human/orphan-designations/eu305268.

3
https://www.ema.europa.eu/en/medicines/human/orphan-designations/eu3161617.

4
https://www.ema.europa.eu/en/medicines/human/EPAR/orphacol.

5 21 C.F.R. § 312.305.

6
https://www.fiercebiotech.com/biotech/biogen-holds-firm-denying-compassionate-use-for-experimental-als-drug.

7
https://www.gov.uk/guidance/apply-for-the-early-access-to-medicines-scheme-eams#fees.
